# Narrow environmental niches predict land-use responses and vulnerability of land snail assemblages

**DOI:** 10.1186/s12862-020-01741-1

**Published:** 2021-02-01

**Authors:** Katja Wehner, Carsten Renker, Nadja K. Simons, Wolfgang W. Weisser, Nico Blüthgen

**Affiliations:** 1 Ecological Networks, Technische Universität Darmstadt, Schnittspahnstraße 3, 64287 Darmstadt, Germany; 2 Naturhistorisches Museum Mainz, Landessammlung für Naturkunde RLP, Reichklarastraße 1, 55116 Mainz, Germany; 3 Department of Ecology and Ecosystem management, Technische Universität München, Hans-Carl-von-Carlowitz-Platz 2, 85350 Freising-Weihenstephan, Germany

**Keywords:** Gastropoda, Land snails, Land-use intensity, Biodiversity Exploratories, Forests, Grasslands

## Abstract

**Background:**

How land use shapes biodiversity and functional trait composition of animal communities is an important question and frequently addressed. Land-use intensification is associated with changes in abiotic and biotic conditions including environmental homogenization and may act as an environmental filter to shape the composition of species communities. Here, we investigated the responses of land snail assemblages to land-use intensity and abiotic soil conditions (pH, soil moisture), and analyzed their trait composition (shell size, number of offspring, light preference, humidity preference, inundation tolerance, and drought resistance). We characterized the species’ responses to land use to identify ‘winners’ (species that were more common on sites with high land-use intensity than expected) or ‘losers’ of land-use intensity (more common on plots with low land-use intensity) and their niche breadth. As a proxy for the environmental ‘niche breadth’ of each snail species, based on the conditions of the sites in which it occurred, we defined a 5-dimensional niche hypervolume. We then tested whether land-use responses and niches contribute to the species’ potential vulnerability suggested by the Red List status.

**Results:**

Our results confirmed that the trait composition of snail communities was significantly altered by land-use intensity and abiotic conditions in both forests and grasslands. While only 4% of the species that occurred in forests were significant losers of intensive forest management, the proportion of losers in grasslands was much higher (21%). However, the species’ response to land-use intensity and soil conditions was largely independent of specific traits and the species’ Red List status (vulnerability). Instead, vulnerability was only mirrored in the species’ rarity and its niche hypervolume: threatened species were characterized by low occurrence in forests and low occurrence and abundance in grasslands and by a narrow niche quantified by land-use components and abiotic factors.

**Conclusion:**

Land use and environmental responses of land snails were poorly predicted by specific traits or the species’ vulnerability, suggesting that it is important to consider complementary risks and multiple niche dimensions.

## Background

Land use disturbs natural environments, changes local geographical landscape structure and alters local biotic and abiotic conditions, e.g. microclimate [1–6]. Reduction of habitat and microhabitat heterogeneity may lead to a homogenization of plant and animal communities, trigger a reduction in functional diversity and thus lower the capacity of an ecosystem to buffer disturbances [7, 8]. Homogenization of animal communities by increasing land-use intensity has been shown for several taxa; e.g., in managed grasslands, 34% of plant- and leafhoppers species were significant losers (i.e. species that were significantly less abundant under conditions of high land-use intensity) of land-use intensification, particularly increases in mowing frequency had a negative effect [9].

Land snails are an important macroinvertebrate group that is directly and indirectly involved in ecosystem processes such as litter decomposition or nutrient cycling [10, 11]. There is a natural north–south and west–east gradient of snail species distributions and abundances within Europe; species richness increases from north to south and to a lesser extent from west to east which is linked to regional and ecological differences and the land-use history [12]. Snail species also differ in their tolerance to abiotic factors (pH, soil moisture), and vary greatly in life-history parameters (e.g., lifespan, development, number of offspring, food requirement, shell size) and general behavior [13] which also affect their distribution. Variation in body size and diet seems to be especially important for structuring snail communities [14] as well as the species-specific tolerance to a variety of environmental factors which can result in nested communities at a specific site [15, 16].

Studies on trait composition of snail communities in Sweden pointed to the importance of the species’ niche-width and the importance of local environmental conditions over spatial variables [17]. While tolerance-related traits such as humidity preference or inundation tolerance were positively associated with abiotic soil moisture, a large amount of variation remained unexplained [17], which may be related to land use. The impact of land use and its intensity on land snail communities is less intensively investigated although most land snail species are characterized by a limited mobility and therefore are vulnerable to human introduced habitat changes [15, 18–20]. Changes in abiotic factors such as soil pH, soil moisture, soil calcium content, leaf litter depth, soil surface structure or the type of vegetation have been shown to alter snail communities [15, 21–25]. Also land-use factors such as the proportion of wood harvested in forests or the amount of grazing livestock in grasslands can influence snail communities directly and/or indirectly [20, 26, 27]. In addition, disturbances by different land-use types and intensities may alter the trait composition of snail communities on the regional level; i.e. the presence of coniferous timber may favor snail communities with differing traits than communities in natural deciduous stands.

In the present study, we investigated land snail communities at forest and grassland sites in different regions of Germany, which were characterized by different land-use types and intensities. We aimed to test whether the trait composition of the snail community is influenced by land-use intensity (and soil conditions). We then tested the responses of each snail species to land-use intensity; ‘winners’ significantly increase in abundance and occurrence with land-use intensity, whereas ‘losers’ significantly decrease compared to the null model [9, 39]. We than compared these responses with the snail species’ habitat association; i.e. we asked whether species that only occasionally occur in forests are more affected by forest management than species that are specialized to forest habitats. On the other hand, do species that are grassland specialist suffer less from grassland management than those only occasionally occurring in grasslands? Finally, we compared our findings of the land-use effects and the ‘winner/loser’ status of a species with its putative vulnerability (Red List status), to test if losers of land-use intensifications in forests and grasslands are those species that are classified as vulnerable.

## Results

### Response to land use

**Fig. 1:**
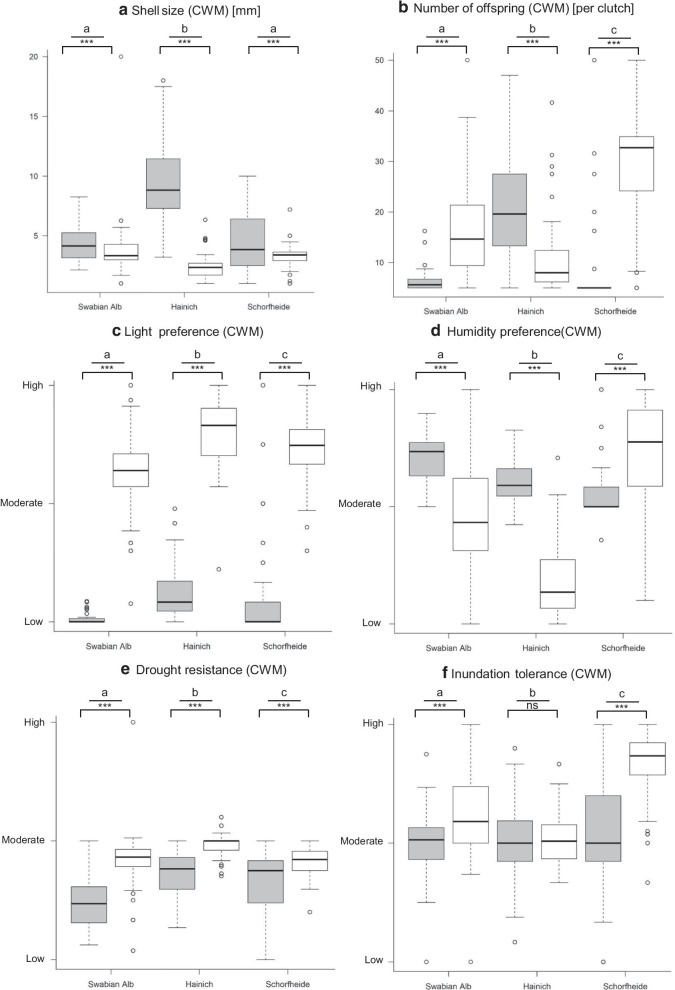
Trait distribution (**a** shell size, **b** number of offspring, **c** light preference, **d** humidity preference, **e** drought resistance, **f** inundation tolerance) of snail communities among forest (grey) and grassland (white) habitats in the Swabian Alb, the Hainich-Dün and the Schorfheide-Chorin. Traits are given as community weighted mean (CWM). Difference among habitats per region are tested using an ANOVA (asterisks), differences between regions are tested by a posthoc Tukey test (letters). Significances: *ns* not significant, *p < 0.05, **p < 0.01, ***p < 0.001

The trait composition of land snail communities differed strongly between forests and grasslands within regions, indicated by a strong differentiation of community-weighted mean trait values (CWMs). Assemblages of forest species consisted of larger species, consistently showed lower light and higher humidity preference, lower drought resistance and mostly lower inundation tolerance than grassland assemblages; differences in the number of offspring were inconsistent among forest and grassland habitats (Fig. 1).

In forests, land-use intensity and abiotic conditions significantly influenced the CWMs of all traits investigated, although often in a different way across regions (Table 1, Additional file 1: Appendix 1; see interaction terms with region). Similarly, in grasslands the trait composition of snail communities was significantly influenced by most land-use components and abiotic conditions (Table 2, Additional file 1: Appendix 1).

**Table 1:** Influence of land-use parameter and abiotic factors on the trait composition of snail communities in forest habitats

Shell size (CWM)	df	Sum Sq	F	p	Number of offspring (CWM)	df	Sum Sq	F	p	Light preference (CWM)	df	Sum Sq	F	p
FORMI	1	457.3	75.683	** < 0.001**	FORMI	1	4692	101.500	** < 0.001**	FORMI	1	0.447	9.763	**0.002**
Region	2	5243.8	433.889	** < 0.001**	Region	2	46,655	504.612	** < 0.001**	Region	2	9.859	107.554	** < 0.001**
FORMI:Region	2	285.4	23.619	** < 0.001**	FORMI:Region	2	753	8.142	** < 0.001**	FORMI:Region	2	1.655	18.055	** < 0.001**
Inonat	1	326.4	54.282	** < 0.001**	Inonat	1	2805	66.118	** < 0.001**	Inonat	1	0.067	1.506	0.220
Region	2	5435.5	451.940	** < 0.001**	Region	2	49,336	581.538	** < 0.001**	Region	2	10.763	121.683	** < 0.001**
Inonat:Region	2	253.2	21.051	** < 0.001**	Inonat:Region	2	3652	43.046	** < 0.001**	Inonat:Region	2	2.687	30.382	** < 0.001**
Idwcut	1	337.7	55.324	** < 0.001**	Idwcut	1	3128	66.613	** < 0.001**	Idwcut	1	0.229	4.818	**0.028**
Region	2	5351.8	438.393	** < 0.001**	Region	2	48,101	512.182	** < 0.001**	Region	2	9.807	102.981	** < 0.001**
Idwcut:Region	2	238	19.495	** < 0.001**	Idwcut:Region	2	166	1.766	0.172	Idwcut:Region	2	0.195	2.048	0.130
Iharv	1	82.4	13.347	** < 0.001**	Iharv	1	1772	37.711	** < 0.001**	Iharv	1	0.948	19.836	** < 0.001**
Region	2	5601.9	453.818	** < 0.001**	Region	2	49,503	526.638	** < 0.001**	Region	2	9.056	94.739	** < 0.001**
Iharv:Region	2	177.2	14.358	** < 0.001**	Iharv:Region	2	80	0.846	0.429	Iharv:Region	2	0.052	0.026	0.580
pH	1	9	1.509	0.220	pH	1	121	2.713	0.099	pH	1	1.523	32.624	** < 0.001**
Region	2	5852.2	489.548	** < 0.001**	Region	2	52,165	583.257	** < 0.001**	Region	2	8.665	92.792	** < 0.001**
pH:Region	2	298.8	24.994	** < 0.001**	pH:Region	2	2091	23.379	** < 0.001**	pH:Region	2	1.410	15.098	** < 0.001**
Soil moisture	1	181.3	28.714	** < 0.001**	Soil moisture	1	1112	23.797	** < 0.001**	Soil moisture	1	0.269	5.600	**0.018**
Region	2	5584.7	442.215	** < 0.001**	Region	2	50,664	541.944	** < 0.001**	Region	2	9.865	102.744	** < 0.001**
Soil mositure:Region	2	55.4	5.261	**0.005**	Soil mositure:Region	2	637	6.811	**0.001**	Soil mositure:Region	2	0.189	1.964	0.141

Humidity preference (CWM)	df	Sum Sq	F	p	Drought resistance (CWM)	df	Sum Sq	F	p	Inundation tolerance (CWM)	df	Sum Sq	F	p
FORMI	1	1.345	37.837	** < 0.001**	FORMI	1	3.511	73.221	** < 0.001**	FORMI	1	2.348	25.064	** < 0.001**
Region	2	12.584	177.015	** < 0.001**	Region	2	14.036	146.369	** < 0.001**	Region	2	1.250	6.670	** < 0.001**
FORMI:Region	2	2.780	34.106	** < 0.001**	FORMI:Region	2	1.589	16.574	** < 0.001**	FORMI:Region	2	1.340	7.151	** < 0.001**
Inonat	1	0.228	6.626	**0.010**	Inonat	1	1.554	33.232	** < 0.001**	Inonat	1	1.603	17.021	** < 0.001**
Region	2	14.311	208.013	** < 0.001**	Region	2	16.025	171.359	** < 0.001**	Region	2	0.969	5.143	**0.006**
Inonat:Region	2	3.280	47.672	** < 0.001**	Inonat:Region	2	2.709	28.963	** < 0.001**	Inonat:Region	2	1.870	9.923	** < 0.001**
Idwcut	1	1.690	46.127	** < 0.001**	Idwcut	1	3.426	72.625	** < 0.001**	Idwcut	1	1.563	16.725	** < 0.001**
Region	2	12.371	168.776	** < 0.001**	Region	2	14.773	156.584	** < 0.001**	Region	2	1.047	5.603	**0.004**
Idwcut:Region	2	1.578	21.527	** < 0.001**	Idwcut:Region	2	1.686	17.868	** < 0.001**	Idwcut:Region	2	2.558	13.687	** < 0.001**
Iharv	1	0.934	24.602	** < 0.001**	Iharv	1	1.122	22.890	** < 0.001**	Iharv	1	0.634	6.904	**0.009**
Region	2	12.937	170.392	** < 0.001**	Region	2	16.400	167.238	** < 0.001**	Region	2	1.246	6.785	**0.001**
Iharv:Region	2	0.493	6.492	**0.002**	Iharv:Region	2	0.560	5.714	**0.003**	Iharv:Region	2	4.873	26.525	** < 0.001**
pH	1	2.750	75.899	** < 0.001**	pH	1	0.470	9.739	**0.002**	pH	1	1.051	11.325	** < 0.001**
Region	2	11.270	155.513	** < 0.001**	Region	2	17.439	180.564	** < 0.001**	Region	2	3.874	20.883	** < 0.001**
pH:Region	2	1.948	26.884	** < 0.001**	pH:Region	2	0.801	8.293	** < 0.001**	pH:Region	2	0.975	5.257	**0.005**
Soil moisture	1	2.921	79.396	** < 0.001**	Soil moisture	1	0.260	5.345	**0.021**	Soil moisture	1	1.966	20.535	** < 0.001**
Region	2	11.494	156.227	** < 0.001**	Region	2	17.561	180.452	** < 0.001**	Region	2	0.125	0.654	0.520
Soil mositure:Region	2	1.020	13.867	** < 0.001**	Soil mositure:Region	2	0.529	5.441	**0.004**	Soil mositure:Region	2	0.935	4.881	**0.008**

Significant values are given in bold

*FORMI* forest management index, *Inonat* proportion of non-native trees, *Idwcut* proportion of dead wood with saw cuts, *Iharv* proportion of wood harvested

**Table 2:** Influence of land-use parameter and abiotic factors on the trait composition of snail communities in grassland habitats

Shell size (CWM)	df	Sum Sq	F	p	Number of offspring (CWM)	df	Sum Sq	F	p	Light preference (CWM)	df	Sum Sq	F	p
LUI	1	5.600	4.283	**0.039**	LUI	1	767	10.237	**0.001**	LUI	1	0.412	5.119	**0.024**
Region	2	187.310	71.659	** < 0.001**	Region	2	35,030	233.853	** < 0.001**	Region	2	17.237	107.017	** < 0.001**
LUI:Region	2	4.300	1.647	0.193	LUI:Region	2	4368	29.160	** < 0.001**	LUI:Region	2	0.103	0.640	0.527
Mowing	1	1.020	0.781	0.377	Mowing	1	14	0.175	0.675	Mowing	1	0.080	1.014	0.314
Region	2	189.840	72.796	** < 0.001**	Region	2	35,805	227.152	** < 0.001**	Region	2	17.245	109.120	** < 0.001**
Mowing:Region	2	8.740	3.350	**0.036**	Mowing:Region	2	1301	8.252	** < 0.001**	Mowing:Region	2	1.605	10.158	** < 0.001**
Grazing	1	0.510	0.389	0.533	Grazing	1	2185	30.582	** < 0.001**	Grazing	1	1.719	22.760	** < 0.001**
Region	2	187.060	71.464	** < 0.001**	Region	2	38,316	268.169	** < 0.001**	Region	2	17.080	113.050	** < 0.001**
Grazing:Region	2	8.230	3.146	**0.044**	Grazing:Region	2	2356	16.489	** < 0.001**	Grazing:Region	2	2.836	18.770	** < 0.001**
Fertilization	1	8.840	6.763	**0.009**	Fertilization	1	2672	33.505	** < 0.001**	Fertilization	1	1.278	15.814	** < 0.001**
Region	2	189.430	70.534	** < 0.001**	Region	2	33,070	207.321	** < 0.001**	Region	2	16.131	99.785	** < 0.001**
Fertilization:Region	2	3.620	1.385	0.251	Fertilization:Region	2	645	4.042	**0.018**	Fertilization:Region	2	0.172	0.690	0..502
pH	1	2.120	1.664	0.197	pH	1	8636	140.899	** < 0.001**	pH	1	0.549	6.939	0.088
Region	2	216.910	85.099	** < 0.001**	Region	2	35,255	287.605	** < 0.001**	Region	2	16.624	105.097	** < 0.001**
pH:Region	2	3.430	1.348	0.261	pH:Region	2	6862	55.976	** < 0.001**	pH:Region	2	1.704	10.771	** < 0.001**
Soil moisture	1	0.490	0.488	0.540	Soil moisture	1	15,105	216.410	** < 0.001**	Soil moisture	1	0.360	4.478	**0.035**
Region	2	190.300	95.150	** < 0.001**	Region	2	24,423	174.950	** < 0.001**	Region	2	16.903	104.523	** < 0.001**
Soil mositure:Region	2	11.580	5.791	**0.012**	Soil mositure:Region	2	4604	32.983	** < 0.001**	Soil mositure:Region	2	0.235	1.453	0.234

Humidity preference (CWM)	df	Sum Sq	F	p	Drought resistance (CWM)	df	Sum Sq	F	p	Inundation tolerance (CWM)	df	Sum Sq	F	p
LUI	1	2.651	14.988	** < 0.001**	LUI	1	0.002	0.082	0.774	LUI	1	1.765	25.385	** < 0.001**
Region	2	123.421	348.964	** < 0.001**	Region	2	2.844	73.056	** < 0.001**	Region	2	43.230	310.828	** < 0.001**
LUI:Region	2	9.841	27.882	** < 0.001**	LUI:Region	2	0.358	9.192	** < 0.001**	LUI:Region	2	3.626	26.071	** < 0.001**
Mowing	1	0.198	1.050	0.306	Mowing	1	0.072	3.703	0.055	Mowing	1	0.401	5.486	**0.019**
Region	2	125.799	333.045	** < 0.001**	Region	2	2.858	73.285	** < 0.001**	Region	2	44.594	304.664	** < 0.001**
Mowing:Region	2	0.582	1.542	0.215	Mowing:Region	2	0.250	6.403	**0.001**	Mowing:Region	2	0.789	5.392	**0.004**
Grazing	1	0.343	1.866	0.172	Grazing	1	0.623	32.926	** < 0.001**	Grazing	1	0.397	5.960	**0.015**
Region	2	128.553	349.785	** < 0.001**	Region	2	2.719	71.585	** < 0.001**	Region	2	46.61	349.538	** < 0.001**
Grazing:Region	2	1.652	4.496	**0.011**	Grazing:Region	2	0.295	7.799	** < 0.001**	Grazing:Region	2	3.843	28.818	** < 0.001**
Fertilization	1	3.182	16.989	** < 0.001**	Fertilization	1	0.016	0.810	0.368	Fertilization	1	4.327	59.483	** < 0.001**
Region	2	122.809	327.885	** < 0.001**	Region	2	2.870	72.506	** < 0.001**	Region	2	41.199	283.187	** < 0.001**
Fertilization:Region	2	1.825	4.872	**0.008**	Fertilization:Region	2	0.067	1.690	0.185	Fertilization:Region	2	0.604	4.152	**0.016**
pH	1	4.880	31.830	** < 0.001**	pH	1	0.111	5.575	**0.018**	pH	1	1.236	20.306	** < 0.001**
Region	2	131.880	430.070	** < 0.001**	Region	2	2.726	68.779	** < 0.001**	Region	2	44.982	369.382	** < 0.001**
pH:Region	2	17.468	56.964	** < 0.001**	pH:Region	2	0.093	2.337	0.097	pH:Region	2	9.133	74.999	** < 0.001**
Soil moisture	1	43.980	258.049	** < 0.001**	Soil moisture	1	0.496	25.671	** < 0.001**	Soil moisture	1	19.377	287.467	** < 0.001**
Region	2	93.991	275.743	** < 0.001**	Region	2	2.497	64.560	** < 0.001**	Region	2	30.888	229.127	** < 0.001**
Soil mositure:Region	2	2.945	8.640	**0.001**	Soil mositure:Region	2	0.312	8.055	** < 0.001**	Soil mositure:Region	2	0.018	0.131	0.877

Significant values are given in bold

*LUI* land-use intensity

In forest habitats, some 4% of all species were ‘losers’ of the combined forest management index (i.e. they were significantly less common in intensively used forests), whereas 12% were ‘winners’ and thus increased with forest management intensity (Table 3). The proportions of non-native trees (4% losers vs. 8% winners) and the proportion of dead wood with saw cuts (6% losers vs. 8% winners) revealed a similar pattern, but for the proportion of wood harvested the percentage of losers (12%) exceeded that of winners (8%).

**Table 3:** Red list status, occurrence and total abundance of snail species in the Swabian Alb (A), the Hainich-Dün (H) and the Schorfheide-Chorin (S) in forest habitats

Species	RedList	Region	Occurrence	Total abundance	FORMI	Inonat	Idwcut	Iharv	pH	Soil moisture
*Acanthinula aculeata* (O.F. Müller, 1774)	*	AHS	37	61	Neutral	Neutral	Neutral	Neutral	Neutral	Neutral
*Aegopinella nitens* (Michaud, 1831)	*	AHS	62	123	**Winner**	Neutral	Neutral	Neutral	Neutral	Neutral
*Aegopinella nitidula* (Draparnaud, 1805)	*	AH	11	15	Neutral	Neutral	Neutral	**Loser**	Mid-specialist	Mid-specialist
*Aegopinella pura* (Alder, 1831)	*	AHS	91	422	Neutral	Neutral	Neutral	Neutral	**"High"**	**"High"**
*Arianta arbustorum* (Linnaeus, 1758)	*	AH	14	24	Neutral	Neutral	**Loser**	Neutral	Neutral	Neutral
*Carychium minimum* O.F. Müller, 1774	*	AH	37	115	**Winner**	Neutral	**Winner**	Neutral	**"High"**	Neutral
*Carychium tridentatum* (Risso, 1826)	*	AHS	74	612	Neutral	Neutral	Neutral	Neutral	**"High"**	**"High"**
*Cecilioides acicula* (O.F. Müller, 1774)	*	AHS	4	4	Neutral	Neutral	Neutral	**Loser**	Neutral	**"Low"**
*Cepaea hortensis* (O.F. Müller, 1774)	*	AH	26	82	**Loser**	**Loser**	**Loser**	**Loser**	**"Low"**	Neutral
*Cepaea nemoralis* (Linnaeus, 1758)	*	H	15	57	Neutral	Neutral	Neutral	Neutral	Neutral	Neutral
*Clausilia bidentata* (Strom, 1765)	*	HS	12	14	Neutral	Neutral	Neutral	Neutral	Neutral	Neutral
*Cochlicopa lubrica* (O.F. Müller, 1774)	*	AHS	29	62	**Winner**	Neutral	**Winner**	**Winner**	**"High"**	Neutral
*Cochlicopa lubricella* (Porro, 1838)	V	A	1	2	Neutral	Neutral	Neutral	Neutral	Neutral	Neutral
*Cochlodina laminata* (Montagu, 1803)	*	AH	19	27	Neutral	Neutral	Neutral	Neutral	Neutral	Neutral
*Discus rotundatus* (O.F. Müller, 1774)	*	AHS	97	362	Neutral	Neutral	Neutral	Neutral	Neutral	Neutral
*Ena montana* (Draparnaud, 1801)	V	AH	10	12	Mid-specialist	Neutral	Neutral	Neutral	**"High"**	Neutral
Euconulus fulvus (O.F. Müller, 1774)	*	AHS	52	86	Neutral	Neutral	Mid-specialist	Neutral	**"Low"**	**"Low"**
*Euomphalia strigella* (Draparnaud, 1801)	G	A	1	1	Neutral	Neutral	Neutral	Neutral	Neutral	Neutral
*Helicodonta obvoluta* (O.F. Müller, 1774)	*	AH	27	77	**Loser**	Neutral	**Loser**	**Loser**	Neutral	Neutral
*Helix pomatia* Linnaeus, 1858	*	H	24	87	**Winner**	**Winner**	**Winner**	**Winner**	Neutral	Neutral
*Isognomostoma isognomostomos* (Schröter, 1784)	*	A	3	3	Neutral	Neutral	Neutral	Neutral	Neutral	Neutral
*Macrogastra plicatul*a (Draparnaud, 1801)	V	A	1	1	Neutral	Neutral	Neutral	**Winner**	Neutral	Neutral
*Macrogastra ventricosa* (Draparnaud, 1801)	*	AH	5	6	Neutral	Neutral	Neutral	Neutral	Neutral	Neutral
*Monacha cartusiana* O.F. Müller, 1774	*	H	2	2	Neutral	Neutral	**Winner**	Neutral	Neutral	Neutral
*Monachoides incarnatus* O.F. Müller, 1774	*	AH	46	118	Neutral	Neutral	Neutral	Neutral	Neutral	Neutral
*Nesovitrea hammonis* (Strom, 1765)	*	AHS	59	169	**Winner**	**Winner**	Neutral	**Loser**	**"Low"**	**"Low"**
*Nesovitrea petronella* (L. Pfeiffer, 1853)	2	S	1	4	Neutral	Neutral	Neutral	Neutral	Neutral	Neutral
*Oxychilus cellarius* (O.F. Müller, 1774)	*	H	7	12	Neutral	Neutral	Neutral	Neutral	Neutral	Neutral
*Oxychilus draparnaudi* (Beck, 1837)	*	H	11	15	Neutral	Neutral	Neutral	**Winner**	Neutral	Neutral
*Platyla polita* (Hartmann, 1840)	3	AH	10	23	Neutral	**Loser**	Neutral	Neutral	Neutral	Neutral
*Punctum pygmaeum* (Draparnaud, 1801)	*	AHS	50	180	Neutral	Neutral	Neutral	Neutral	Neutral	**"Low"**
*Pupilla muscorum* (Linnaeus, 1758)	V	HS	2	12	Neutral	Neutral	Neutral	Neutral	Neutral	Neutral
*Succinella oblonga* (Draparnaud, 1801)	*	A	2	2	Neutral	Neutral	Neutral	Neutral	Neutral	Neutral
*Trochulus hispidus* (Linnaeus, 1758)	*	AH	11	16	Neutral	Neutral	Neutral	Neutral	Neutral	Neutral
*Trochulus sericeus* (Draparnaud, 1801)	*	AH	8	12	Neutral	Neutral	Neutral	Neutral	Neutral	Neutral
*Trochulus striolatus* (C. Pfeiffer, 1828)	V	A	16	25	Neutral	Neutral	Neutral	Neutral	Neutral	**"Low"**
*Urticicola umbrosus* (C. Pfeiffer, 1828)	V	S	1	1	Neutral	Neutral	Neutral	Neutral	Neutral	Neutral
*Vallonia costata* (O.F. Müller, 1774)	*	AS	2	3	Neutral	Neutral	Neutral	Neutral	Neutral	Neutral
*Vallonia excentrica* Sterki, 1893	*	AH	7	25	Neutral	Neutral	Neutral	**Loser**	Neutral	Neutral
*Vallonia pulchella* (O.F. Müller, 1774)	*	AHS	14	38	Neutral	Neutral	Neutral	Neutral	**"Low"**	**"Low"**
*Vertigo angustior* Jeffreys, 1830	3	A	1	1	Neutral	**Winner**	Neutral	Neutral	Neutral	Neutral
*Vertigo pygmaea* (Draparnaud, 1801)	*	HS	3	9	Neutral	Neutral	Neutral	Neutral	Neutral	Neutral
*Vertigo substriata* (Jeffreys, 1833)	3	AS	2	3	Neutral	Neutral	Neutral	Neutral	Neutral	Neutral
*Vitrea contracta* (Westerlund, 1871)	*	AHS	40	89	Neutral	Neutral	Neutral	Neutral	**"High"**	Neutral
*Vitrea crystallina* (O.F. Müller, 1774)	*	AH	16	30	Neutral	Neutral	Neutral	Mid-specialist	Neutral	Neutral
*Vitrea diaphana* (Studer, 1820)	G	H	11	27	Neutral	Neutral	Neutral	Neutral	Neutral	Neutral
*Vitrina pellucida* (O.F. Müller, 1774)	*	S	1	1	**Winner**	Neutral	Neutral	Neutral	Neutral	Neutral
*Vitrinobrachium breve* (A. Férussac, 1821)	*	A	2	2	Neutral	Neutral	Neutral	Neutral	**"Low"**	Neutral
*Zonitoides nitidus* (O.F. Müller, 1774)	*	AS	2	2	Neutral	**Winner**	Neutral	Neutral	Neutral	Neutral

Significant values are given in bold

Species responses to land use are assigned as winner, loser or mid-specialist to the following land-use parameters forest management index (FORMI), the percentage of non-native trees (Inonat), the percentage of dead wood with saw cuts (Idwcut) and the percentage of tree harvesting (Iharv)

“Low” and “high” refer to low- and high-gradient species, respectively. Red List status: * = no current risk (least concern), G = endangered to unknown extent, R = very rare, V = near threatened, 1 = critically endangered, 2 = endangered, 3 = vulnerable

In grasslands, many species were predominantly found at low land-use intensities (LUI); 21% of all species were significant losers and only *Monacha cartusiana* profited from high LUI (Table 4). However, single land-use components in grasslands had only weak effects. Grazing intensity positively affected *Cecilioides acicula* and *Cepaea hortensis*, but showed no negative impact. Similarly, mowing (2% losers and 2% winners) and fertilization (4% losers and 4% winners) had a very little impact compared to the combined LUI.

**Table 4:** Red list status, occurrence and total abundance of snail species in the Swabian Alb (A), the Hainich-Dün (H) and the Schorfheide-Chorin (S) in grassland habitats

Species	RedList	Region	Occurrence	Total abundance	LUI	Grazing	Mowing	Fertilization	pH	Soil moisture
*Abida secale* (Draparnaud, 1801)	G	A	1	1	Neutral	Neutral	Neutral	Neutral	Neutral	Neutral
*Acanthinula aculeata* (O.F. Müller, 1774)	*	AH	2	2	Neutral	Neutral	Neutral	Neutral	Neutral	Neutral
*Aegopinella nitens* (Michaud, 1831)	*	AHS	9	15	**Loser**	Neutral	Neutral	Neutral	Neutral	Neutral
*Aegopinella nitidula* (Draparnaud, 1805)	*	H	1	2	Neutral	Neutral	**Winner**	Neutral	Neutral	Neutral
*Aegopinella pura* (Alder, 1831)	*	AH	21	38	Neutral	Neutral	Neutral	Neutral	Neutral	Neutral
*Candidula unifasciata* (Poiret, 1801)	2	AH	9	46	Neutral	Neutral	Neutral	Neutral	**"High"**	**"Low"**
*Carychium minimum* O.F. Müller, 1774	*	AS	23	381	Neutral	Neutral	Neutral	**Loser**	**"High"**	**"High"**
*Carychium tridentatum* (Risso, 1826)	*	AHS	30	142	Neutral	Neutral	Neutral	Neutral	**"High"**	**"High"**
*Cecilioides acicula* (O.F. Müller, 1774)	*	AH	2	2	Neutral	**Winner**	Neutral	Neutral	Neutral	Neutral
*Cepaea hortensis* (O.F. Müller, 1774)	*	AH	3	3	Neutral	**Winner**	**Loser**	Neutral	Neutral	Neutral
*Cochlicopa lubrica* (O.F. Müller, 1774)	*	AHS	77	546	Neutral	Neutral	Neutral	Neutral	Neutral	Neutral
*Cochlodina laminata* (Montagu, 1803)	*	H	1	1	Neutral	Neutral	Neutral	Neutral	Neutral	Neutral
*Columella aspera* Waldén, 1966	*	A	1	1	Neutral	Neutral	Neutral	Neutral	Neutral	Neutral
*Discus rotundatus* (O.F. Müller, 1774)	*	AHS	12	28	Neutral	Neutral	Neutral	**Winner**	Neutral	**"Low"**
*Eucobresia diaphana* (Draparnaud, 1805)	*	H	1	1	Neutral	Neutral	Neutral	Neutral	Neutral	Neutral
Euconulus fulvus (O.F. Müller, 1774)	*	S	1	1	Neutral	Neutral	Neutral	Neutral	Neutral	Neutral
*Euomphalia strigella* (Draparnaud, 1801)	G	A	1	1	Neutral	Neutral	Neutral	Neutral	Neutral	Neutral
*Granaria frumentum* (Draparnaud, 1801)	2	A	2	18	**Loser**	Neutral	Neutral	Neutral	**"High"**	Neutral
*Helicella itala* (Linnaeus, 1858)	3	AH	11	28	**Loser**	Neutral	Neutral	Neutral	**"High"**	Neutral
*Helicodonta obvoluta* (O.F. Müller, 1774)	*	A	1	1	Neutral	Neutral	Neutral	Neutral	Neutral	Neutral
*Helix pomatia* Linnaeus, 1858	*	H	3	6	Neutral	Neutral	Neutral	Neutral	Mid-specialist	Neutral
*Macrogastra ventricosa* (Draparnaud, 1801)	*	AH	3	3	Neutral	Neutral	Neutral	Neutral	Neutral	Neutral
*Monacha cartusiana* O.F. Müller, 1774	*	HS	2	34	**Winner**	Neutral	Neutral	Neutral	Neutral	Neutral
*Monachoides incarnatus* O.F. Müller, 1774	*	AH	3	3	Neutral	Neutral	Neutral	Neutral	Neutral	Neutral
*Nesovitrea hammonis* (Strom, 1765)	*	AHS	16	35	**Loser**	Neutral	Neutral	Neutral	**"Low"**	Neutral
*Oxychilus draparnaudi* (Beck, 1837)	*	A	1	2	Neutral	Neutral	Neutral	**Winner**	Neutral	Neutral
*Platyla polita* (Hartmann, 1840)	3	A	1	1	Neutral	Neutral	Neutral	Neutral	Neutral	Neutral
*Pseudotrichia rubiginosa*	2	S	1	1	Neutral	Neutral	Neutral	Neutral	**"High"**	Neutral
*Punctum pygmaeum* (Draparnaud, 1801)	*	AHS	14	28	**Loser**	Neutral	Neutral	Neutral	Neutral	Neutral
*Pupilla muscorum* (Linnaeus, 1758)	V	AHS	70	1087	Neutral	Neutral	Neutral	Neutral	**"High"**	Neutral
*Pupilla alpicola* (Clessin, 1871)	R	S	12	530	Neutral	Neutral	Neutral	Neutral	Neutral	Neutral
*Succinea putris* Beck, 1837	*	S	13	165	Neutral	Neutral	Neutral	Neutral	Neutral	Neutral
*Succinella oblonga* (Draparnaud, 1801)	*	AHS	25	57	Neutral	Neutral	Neutral	Neutral	Neutral	Mid-specialist
*Trochulus hispidus* (Linnaeus, 1758)	*	AHS	29	215	**Loser**	Neutral	Neutral	Neutral	**"High"**	Neutral
*Trochulus sericeus* (Draparnaud, 1801)	*	AH	9	14	Neutral	Neutral	Neutral	Mid-specialist	Neutral	Neutral
*Trochulus striolatus* (C. Pfeiffer, 1828)	V	A	1	1	Neutral	Neutral	Neutral	Neutral	Neutral	Neutral
*Truncatellina cylindrica* (A. Férussac, 1807)	3	AH	4	14	**Loser**	Neutral	Neutral	Neutral	**"High"**	**"Low"**
*Vallonia costata* (O.F. Müller, 1774)	*	AHS	40	493	Neutral	Neutral	Neutral	Neutral	Mid-specialist	Neutral
*Vallonia enniensis* (Gredler, 1856)	1	AS	5	63	Neutral	Neutral	Neutral	Neutral	**"High"**	Neutral
*Vallonia excentrica* Sterki, 1893	*	AHS	106	1829	Neutral	Neutral	Neutral	Neutral	Neutral	**"Low"**
*Vallonia pulchella* (O.F. Müller, 1774)	*	AHS	96	3456	Neutral	Neutral	Neutral	Neutral	Neutral	**"High"**
*Vertigo angustior* Jeffreys, 1830	3	S	9	47	Neutral	Neutral	Neutral	Neutral	Neutral	Neutral
*Vertigo antivertigo* (Draparnaud, 1801)	V	AS	12	102	Neutral	Neutral	Neutral	**Loser**	**"High"**	Neutral
*Vertigo pygmaea* (Draparnaud, 1801)	*	AHS	69	355	**Loser**	Neutral	Neutral	Neutral	Neutral	**"High"**
*Vertigo substriata* (Jeffreys, 1833)	3	S	1	2	Neutral	Neutral	Neutral	Neutral	Neutral	Neutral
*Vitrea contracta* (Westerlund, 1871)	*	AH	5	8	**Loser**	Neutral	Neutral	Neutral	Neutral	Neutral
*Vitrea diaphana* (Studer, 1820)	G	H	2	2	Neutral	Neutral	Neutral	Neutral	Neutral	Neutral
*Vitrina pellucida* (O.F. Müller, 1774)	*	AHS	10	16	**Loser**	Neutral	Neutral	Neutral	Neutral	Mid-specialist

Significant values are given in bold

Species are assigned as winner, loser or mid-specialist to the following land-use parameters land-use index (LUI), grazing, mowing and fertilization intensity

“Low” and “high” refer to low- and high-gradient species, respectively. Red List status: * = no current risk (least concern), G = endangered to unknown extent, R = very rare, V = near threatened, 1 = critically endangered, 2 = endangered, 3 = vulnerable

However, in both forests and grasslands, species’ land-use responses (i.e. their ‘winner/loser’ status) were independent of their traits; i.e. losers in forests or grasslands were neither characterized by a smaller or larger shell size nor by lower or higher numbers of offspring nor by lower or higher light preference etc. (Additional files 2–15: Appendix 2–15).

### Response to abiotic factors

Although niches of common land snail species for soil pH and soil moisture were generally broad, some differentiation was found in the communities of both habitats. In forests, *Aegopinella pura*, the genus *Carychium*, *Cochlicopa lubrica*, *Ena montana* and *Vitrea contracta* were significantly associated with higher pH values (Table 3) and *Cepaea hortensis*, *Euconulus fulvus*, *Nesovitrea hammonis*, *Vallonia pulchella* and *Vitrinobrachium breve* were found at sites with low pH (Table 3). Furthermore, *A. pura* and *Carychium tridentatum* were associated with high soil moisture in forests and *Ceciliodes acicula*, *E. fulvus*, *N. hammonis*, *Punctum pygmaeum*, *Trochulus striolatus* and *V. pulchella* were found at low soil moisture values (Table 3).

Grassland sites had a higher mean pH (6.7) as compared to forest soils, and many snail species (e.g., *Candidula unifasciata*, the genus *Carychium*, *Granaria frumentum*, *Pupilla muscorum*, *Vertigo antivertigo*) were associated with higher pH values (Table 4). Only *N. hammonis* was significantly more common on sites with low pH. Soil moisture niches of grassland species were even broader than those of pH. The genus *Carychium*, *Trochulus hispidus* and *Vallonia pulchella* were found at high moisture values, while *C. unifasciata*, *Discus rotundatus*, *Truncatellina cylindrica*, *V. excentrica* were associated with low soil moisture (Table 4).

### Habitat association

**Fig. 2:**
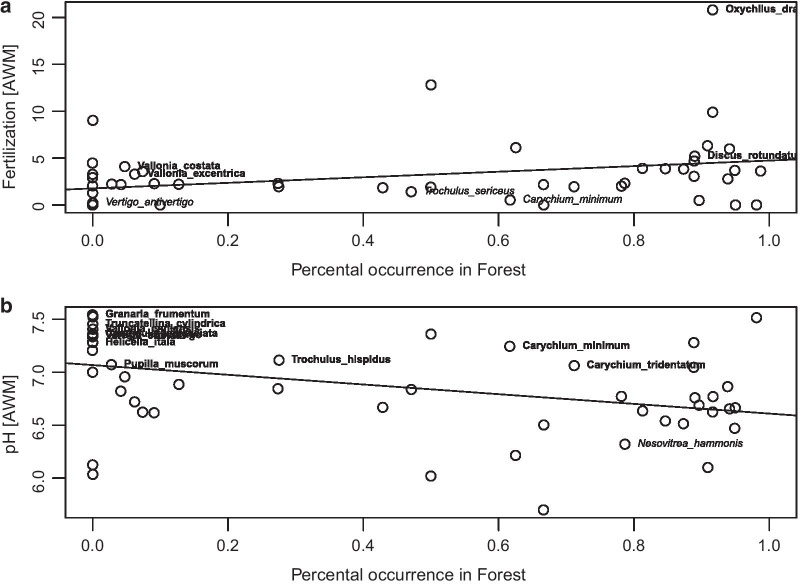
Relation between the responses (abundance-weighted mean) of each snail species to fertilization (**a**) and soil pH (**b**) and their proportional occurrence in forests. Indicated species above the line are significant “winners” for fertilization respective soil pH, indicated species below the line (in italic) are significant “losers”

**Fig. 3:**
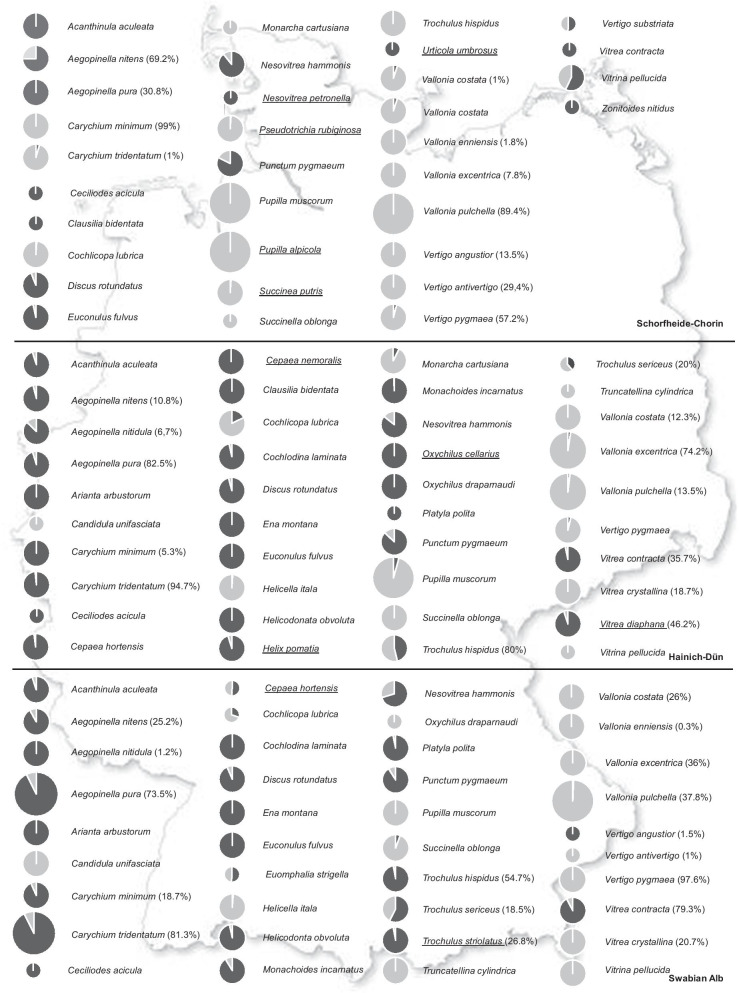
Proportional distribution of land snail species in the Schorfheide-Chorin, the Hainich-Dün and the Swabian Alb. Grasslands are given in light grey, forests in dark grey. The three most abundant species are symbolized by big circles, less abundant species by small circles. Species that are underlined are specific for the respective region. Percentages in brackets indicate the proportional occurrence of species of the same genus

Snail species differed in their habitat association and their distribution among regions (Fig. 2). However, effects of land-use management components and abiotic factors in forests were independent of the species’ habitat association, i.e. species that occurred in forests at low frequencies (e.g., 25% of the individuals in *Cochlicopa lubrica*; Fig. 2) were equally affected by land-use intensification as species that are exclusively found in forests (e.g., *Cepaea hortensis*) (F_1,49_ = 0.14, p = 0.71, Fig. 2, Additional file 14: Appendix 14). In contrast, grassland species that predominately prefer grassland habitats were less tolerant to fertilization than species that also occur in forests (F_1,50_ = 5.84, p = 0.019, Fig. 3a, Additional file 15: Appendix 15). Furthermore, grassland “specialists” were significantly associated with higher pH values (F_1,49_ = 9.21, p = 0.004, Fig. 3b).

### Species’ vulnerability

Across forests and grasslands, 75% of the 61 snail species found are currently not threatened or endangered according to their Red List status (Tables 3, 4). Nevertheless, *Nesovitrea petronella*, *Candidula unifasciata* and *Granaria frumentum* are regarded as ‘endangered’ while *Vallonia enniensis* is ‘highly endangered’ and *V. angustior* is listed on the FFH directive.

**Fig. 4:**
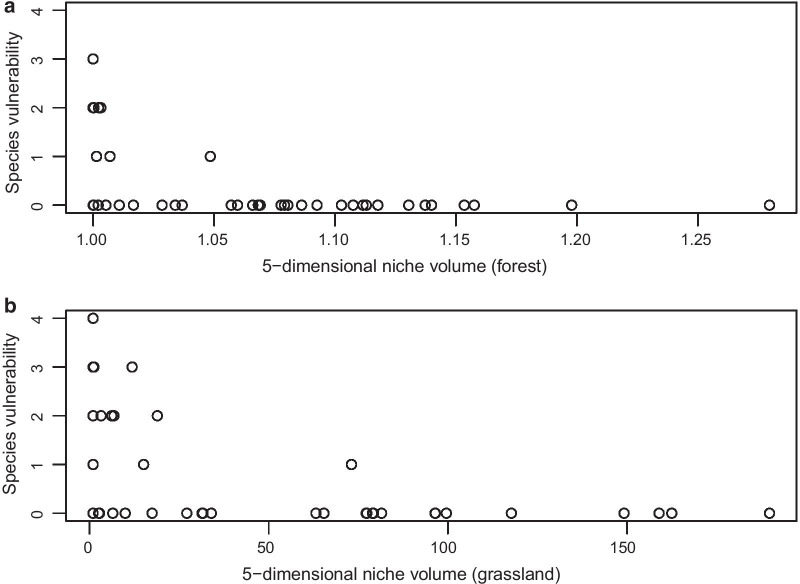
Species vulnerability in relation to the five-dimensional niche hypervolume in forest (**a**) and grassland (**b**). The hypervolume was the product of the abundance-weighted standard deviations (AWSDs) of all single land-use components as well as pH and soil moisture in forests or grasslands, respectively

There was no statistical support that a negative response to land-use intensity of a certain species (“loser”) is associated with a high vulnerability of the species, neither in forests nor in grasslands (Table 5). A better predictor for the species’ vulnerability in forests was a relatively low number of sites in which the species occurred, and in grasslands both a low occurrence and a low total abundance corresponded to a higher vulnerability (Table 5). Furthermore, the 5-dimensional niche hypervolume based on the species’ tolerance to land-use components and abiotic conditions was significantly correlated with the species’ vulnerability, hence species with a small niche hypervolume are more vulnerable in both forests (Spearman rank test: S = 20,091, p = 0.0004; Fig. 4a) and grasslands (Spearman rank test: S = 15,547, p = 0.003, Fig. 4b).

**Table 5:** Statistical p values of a general linearized model with Poisson distribution testing the influence of land-use parameters and abiotic factors on species vulnerability

Species vulnerability	Estimate	p value	Species vulnerability	Estimate	p value
FORMI	− 0.224	0.689	LUI	− 0.511	0.256
Occurrence	− 1.441	**0.002**	Occurrence	− 1.303	** < 0.001**
Total abundance	0.546	0.158	Total abundance	0.673	**0.001**
Inonat	− 0.424	0.150	Mowing	− 0.031	0.903
Occurrence	− 1.512	**0.002**	Occurrence	− 1.227	** < 0.001**
Total abundance	0.598	0.112	Total abundance	0.638	**0.001**
Idwcut	− 0.094	0.945	Grazing	− 0.049	0.339
Occurrence	− 1.454	**0.005**	Occurrence	− 1.212	** < 0.001**
Total abundance	0.573	0.177	Total abundance	0.643	** < 0.001**
Iharv	0.119	0.948	Fertilization	− 0.038	0.413
Occurrence	− 1.477	**0.002**	Occurrence	− 1.224	** < 0.001**
Total abundance	0.594	0.103	Total abundance	0.616	**0.001**
pH	0.198	0.573	pH	0.092	0.849
Occurrence	− 1.643	**0.004**	Occurrence	− 2.001	** < 0.001**
Total abundance	0.699	0.104	Total abundance	0.615	**0.012**
Soil moisture	0.039	0.333	Soil moisture	− 0.043	0.330
Occurrence	− 1.719	**0.002**	Occurrence	− 1.184	** < 0.001**
Total abundance	0.726	0.063	Total abundance	0.631	**0.001**

Significant values are given in bold

## Discussion

### Response to land use and abiotic factors

Land snail species are slow-dispersing organisms, and historical influences are of general importance for their distribution [28]. Their diversity and heterogeneity is modified by predation, parasitism, competition, abiotic environmental gradients, natural barriers and disturbances [16]. While abiotic and vegetation parameters can be used to predict snail communities, disturbances by human land use are less frequently discussed. Our previous study [27] focused on land snail density, diversity and species composition and emphasized that direct impacts of land use on snail communities were on average lower than the impact of abiotic drivers and biotic substrates. However, unlike several studies on insects, few direct effects have been shown for wood harvesting in forests and mowing in grasslands on snail diversity [27]. How these direct land-use effects influence populations of single species and whether these effects are related to species-specific traits remains largely unclear.

Our study showed that snail assemblages varied consistently in their trait composition (shell size, number of offspring, light and humidity preference, drought resistance and inundation tolerance) across regions and among the two habitats, forests and grasslands. The variation between regions is consistent with a biogeographic gradient of increasing land snail diversity from the north to south caused by historical and ecological factors (temperature, moisture) [12, 22] and snail species responded differently to variable physical environments [13]. Local environmental conditions have been shown to explain about 19% of the trait variability of a snail metacommunity in Sweden [17], where the authors suggested that the unexplained variation may mirror land use. Our results confirmed that land-use intensity significantly influenced the trait distribution of snail communities, a pattern that was more pronounced in forest habitats than in grasslands. Since snail species in forest communities seem to be more specialized than those of grassland communities [12, 28], they may suffer more from habitat changes. For example, as the activity level of snails is temperature-dependent, thinning the canopy by wood harvesting or a high amount of non-native trees can enhance solar irradiance and the enhanced snail locomotion allows the exploitation of ambient heterogeneity [29] and may favor species with higher light preferences. This hypothesis is consistent with results from snail assemblages in our study, since the community-weighted mean (CWM) of light preference increased with the amount of non-native, mainly coniferous trees that may not have a closed canopy. Furthermore, changes of the community trait composition are not only directly caused by land-use parameters, but also by indirectly changing abiotic factors such as soil pH and soil moisture although most snail species exhibit broad niches for these abiotic factors.

In our study, 4% of all forest and 21% of all grassland snail species were significant losers concerning the compound indices of land-use intensity, including three land-use components in the forests or in the grasslands, respectively. The proportion of losers among grassland snail species was lower than the level found for grasshoppers (about 52%) [30] and plant- and leafhoppers (about 34%) [9], but similar to that for moths (28%) [31], confirming that snails are a suitable indicator for habitat quality and land-use intensity [17, 22, 32, 33]. The low proportion of loser species may be explained by their ground-living behavior (intangible for combine harvesters), the presence of a shell (protection against exposure and predation) and a larger diet breath compared to insect taxa (omnivory for flexibly changing food resources). However, we may have underestimated the amount of loser species since we did not distinguish between living individuals and empty shells. Empty shells decay at different rates under different ecological conditions [44]. Therefore, in some cases we may have evaluated shells of species which can no longer be found alive in the respective places. Keeping this in mind, our methodological approach may have ramifications on the conclusions drawn.

While increasing land-use intensity in open habitats is known to trigger a decline of pollinator species, and such losses were associated with species-specific trait attributes such as a narrow diet breadth, climate specialization, a large body-size and low fecundity [33–39], we did not find traits for snail species to correspond with their land-use response at species level. This is surprising, given that particularly those traits that are associated with soil moisture (drought resistance, inundation tolerance), body size or reproductive outcome are likely to respond to human-mediated disturbances. Furthermore, land-use effects in forests were independent of the species habitat association (i.e. forests specialists were equally affected as non-forests specialists), but grassland specialists suffered more from land use (i.e. fertilization) and were more dependent on high soil pH.

Note that single land-use parameters and abiotic conditions are often confounded in real landscapes as in our study, and thus responses of some snail species may not always correspond to single environmental dimensions as known from their global distribution or other sources. For example, *Cochlicopa lubricella* is a xerophilic land snail [42] whereas our data showed a neutral response to soil moisture.

### Species’ vulnerability

The range of resources and the ecological conditions generally define the niche breadth and determine the geographical area of a species at the small or large scale [40]. Specialists are expected to be more vulnerable to habitat loss and climate change due to synergistic effects of a narrow niche and a small range size.

Only a few snails in our study across managed forests and grasslands are considered threatened or endangered according to the national Red List. Consistent with the expectation based on their environmental niche breadth, the species’ vulnerability status was significantly predicted by a particularly narrow niche hypervolume—an index that includes single land-use components as well as pH and soil moisture in each habitat. The smaller the hypervolume of a species, the higher its vulnerability according to the Red List. In addition, rarity was important: in forests, the most important predictor for their vulnerable status was a low number of sites in which they occurred. In grasslands, both their restricted occurrence and low total abundance predicted the species’ vulnerability.

## Conclusion

In summary, our results indicate that the trait composition of snail communities was significantly altered by land-use intensities and abiotic conditions, and several species especially in grasslands were losers of intensive land use. These land-use and environmental responses were largely independent of specific traits and the species’ Red List status—this suggests that complementary risks may be important for predicting a species’ vulnerability. Instead, species vulnerability was mirrored in the species’ rarity and its overall niche hypervolume including single land-use components and abiotic factors.

## Methods

### Data origin

Data for this study were already part of a previous analysis of biodiversity and community composition, i.e. Wehner et al. [27] and are available at https://www.bexis.uni-jena.de/PublicData/PublicDataSet.aspx?DatasetId=24986. Wehner et al. [27] collected 15,607 snail individuals belonging to 71 taxa in three regions in Germany in the framework of the Biodiversity Exploratories Project (http://www.biodiversity-exploratories.de) [2]. The collaborative research unit addresses effects of land-use on biodiversity and biodiversity-related ecosystem processes in three regions: the Swabian Alb (ALB), a low-mountain range in South-West Germany (460–860 m a.s.l., 09° 10′ 49″–09° 35′ 54″ E/48° 20′ 28″–48° 32′ 02″ N), the Hainich-Dün (HAI), a hilly region in Central Germany (285–550 m a.s.l., 10° 10′24″–10° 46′ 45″ E/50° 56′ 14″–51° 22′ 43″ N) and the Schorfheide-chorin (SCH), a glacial formed landscape in North-East Germany (3–140 m a.s.l., 13° 23′ 27″–14° 08′ 53″ E/52° 47′ 25″–53° 13′ 26″ N). SCH is characterized by the lowest annual precipitation (520–580 mm), with a mean annual temperature of 6–7 °C. It is followed by HAI (630–800 mm, 6.5–8 °C) and ALB (800–930 mm, 8–8.5 °C).

In each region, 100 experimental plots (50 in forests and 50 in grasslands) were setup in 2008 along a land-use gradient covering different management types and intensities including mowing, grazing and fertilization in grasslands and the proportion of non-native trees, the proportion of dead-wood with saw cuts and the proportion of wood harvested in forests (Table 6). Forest plots have a size of 1 ha and grassland plots are 0.5 ha in size.

**Table 6:** Description and origin of land-use parameter and abiotic factors

Habitat	Land-use parameter	Desciption/unit	Range	References	Dataset ID	Source/owner	Year used
Grassland	Mowing	Frequency per year	0–3	Blüthgen et al. 2012 [41]	19266 version 1.15.12	Katrin Lorenzen	Mean of 2015/2016
	Grazing	Livestock units × days of grazing × ha^−1^ × year^−1^	0–851	Blüthgen et al. 2012 [41]		Wolfgang Weisser	Mean of 2015/2016
	Fertilization	Kg nitrogen × ha^−1^ × year^−1^	0–433	Blüthgen et al. 2012 [41]		Manfred Ayasse	Mean of 2015/2016
	Land-use index LUI	The compound LUI index adds fertilization plus mowing plus grazing intensities. Each individual LUI component (fertilization, mowing and grazing) was standardized relative to its mean within the corresponding model region	0.53–4.52	Blüthgen et al. 2012 [41]		Markus Fischer Juliane Vogt	Mean of 2015/2016
Forest	Proportion of non-native trees	Estimated as the proportion of harvested, living and dead wood volume of non-natural tree species to the sum volume of all tree species	0–1	Kahl and Bauhus 2014 [40]	24646 version 1.2.8	Peter Schall Christian Ammer Jürgen Bauhus	2017
	Proportion of dead-wood with saw cuts	Represents the proportion of dead wood with saw cuts to the total amount of dead wood	0–1	Kahl and Bauhus 2014 [40]			2017
	Proportion of wood harvested	Describes the proportion of harvested tree volume within a stand and is estimated by the presence of cut stumps and calculated as the ratio of harvested volume to the sum of standing, harvested and dead wood volume	0–1	Kahl and Bauhus 2014 [40]			2017
	Forest management index Formi	The Formi is the sum of three components taking into account: 1. the proportion of harvested tree volume, 2. the proportion of tree species that are not part of the natural forest community and 3. the proportion of dead wood showing signs of saw cuts. Each component ranges between 0 (no sign of management) and 1 (intensive management)	0–2.82	Kahl and Bauhus 2014 [40]			2017
Grassland/forest	Soil pH		3.0–6.7		22246 Verion 1.1.9	Ingo Schöning	Mean 2017
						Theresa Klotzing	
						Antonios Apostolakis	
						Susan Trumbore	
	Soil moisture	Soil moisture in 10 cm depth, as percentage of the volumemetric water content	8.55–55.22		Weather station	Marion Schrumpf	Mean May 2017
					Climate tool	Falk Hänsel	
						Stephan Wöllauer	
						Thomas Nauss	

In June 2017, Wehner et al. [27] took five replicated surface samples from all 50 forest and 50 grassland experimental plots (EPs) in the Swabian Alb and the Hainich, and from 49 forest and 34 grassland plots in the Schorf-heide due to constrained accessibility (1415 samples in total). Shelled snails were subsequently determined to the species, genus or family level using [41–43]. Although suggested elsewhere [e.g., 44], [27] did not distinguish between empty shells and living snail individuals.

As our current study focuses on species-level responses, only those individuals that could be assigned to the species level were used (ALB grasslands: 36, ALB forests: 37, HAI grassland: 31, HAI forest: 35, SCH grassland: 24, SCH forest: 21, 61 different land snail species in total). Grassland plots (although not permanently flooded) in one region (Schorfheide) harbored large numbers of aquatic and semi-aquatic snails. In contrast to our previous analysis that covered all snails recorded [27], we excluded aquatic snails from the analyses since their role and responses to terrestrial environmental variables such as land-use in grasslands remain unclear,

### Statistical analyses

All statistical analyses were performed in R 3.5.2 [45] using the main packages “car” [46], “dplyr” [47], “lme4” [48] and “SMDTools” [49].

### Trait composition of snail communities

Morphological and life-history trait values for all snail species were obtained from an established trait database by Falkner et al. [50] and compared to findings of [51] whenever possible; see Astor et al. [17] for a similar approach based on [50]. Traits for the set of species in our study are summarized in Table 7. Note that these traits are either continuous variables (size), integers (offspring) or ranks (all others); ranks can been treated as integers or continuous variables for an analysis based on community weighted mean (CWM, see below); the resulting distribution of the CWM in species-rich communities and across a large number of plots typically approach a Gaussian distribution. Moreover, to explore the response to potential environmental filtering, traits with different meaning are treated independently for the following analysis (a common practice, although some traits, e.g. shell size and number of offspring, may be correlated, see [17]).

**Table 7:** Characterization of snail traits according to Falkner et al. 2001 [50]

Trait	Explanation	Unity
Shell size	Maximal height of an oblong shell or the maximal diameter of a depressed shell in mm; in case of globose/conical shells, whichever measure has the greater value is considered	mm
Number of offspring	Numbers of eggs/juveniles per clutch	1–10, 11–100, > 100
Light preference	Degree to which species occur in direct sunlight or shaded conditions	Deep shade, light shade, no shade, indifferent
Humidity preference	Degree to which species occur at wet or dry conditions	Wet, moist and dry
Drought resistance	Degree to which species can survive dry periods	Hours, days, weeks, months
Inundation tolerance	Degree to which species are tolerant to inundation	Low, moderate, high

For comparing snail communities among habitats and regions, the community weighted mean (CWM) of each trait was calculated as CWM per plot *p*

$${CWM}_{p}= \sum_{i=1}^{I}{T}_{i}\bullet \frac{{a}_{i,p}}{{A}_{p}}$$

where *T*_*i*_ is the trait value of species *i*, *a*_*i,p*_ is the abundance of species *i* in plot *p* and *A*_*p*_ the total abundance of all snails in plot *p* (total *I* species).

### Environmental niches

We characterized the environmental conditions of each forest or grassland plot by its land-use intensity and two abiotic soil parameters (pH and soil moisture; Table 6) [52, 53]. Data were obtained from the BExIS database (Table 6).

We tested the response of the CWM of each trait to variation in environmental conditions using linear regressions. Values for grazing and fertilization were square root transformed before statistical analyses.

In order to characterize the snail species’ responses to environmental conditions (land-use gradient, soil conditions), we calculated each species’ “environmental niche”. The method has been established in the context of the Biodiversity Exploratories and was applied to several taxa such as grasshoppers [30], cicadas, moths [31], bumblebees [54] or plants [55]. The “niche optimum” was calculated as the abundance weighted mean (AWM) for species *i* as

$${AWM}_{i}= \sum_{p=1}^{{n}_{p}}{L}_{p}\bullet \frac{{a}_{i,p}}{{A}_{i}}$$

where *n*_*p*_ is the number of plots investigated, *L*_*p*_ is the land-use gradient value of plot *p*, *a*_*i,p*_ the abundance of species *i* in plot *p* and *A*_*i*_ the total abundance of species i across all 149 forest or 134 grasslands sites, respectively. Hence, the CWM characterizes the plots by the trait distribution of snails, and the AWM characterizes snail species by the environmental conditions of the plot, and the snail abundance *a*_*i,p*_ is used to weight either species or plot, respectively.

In addition to the AWM as a niche optimum, we also characterized the “niche breadth” of each species to a single environmental variable using the abundance-weighted standard deviation (AWSD) [30]. To test whether AWMs and AWSDs statistically deviate from an expected random distribution, we compared the calculated values against the expected values obtained from a null model that distributes each species across *N*_*i*_ sites with the same probability, with *N*_*i*_ being the number of sites in which species *i* was found. The null model thus chooses values of the focal land-use parameter (LUI, Formi, single components, pH, soil moisture) of *N*_*i*_ sites and calculates a distribution of predicted AWMs and AWSDs values for each species based on 10,000 iterations. The null model was restricted to the one, two, or three regions in which the species was recorded to consider potential distribution boundaries of each species in Germany that may not be related to plot conditions [30].

As in any randomization model, the proportion of AWMs or AWSDs from 10,000 null models with greater or smaller expected values respectively than the observed value, provides the *p *value for the significance of the deviation between observed and expected values. A ‘winner’ is defined as a species with an observed AWM larger than the upper 5% of the distribution of AWMs obtained by the null models (i.e. adapted on higher-than average land-use intensity), a ‘loser’ shows an observed AWM smaller than the lower 5% (low land-use intensity specialist). For species which could be classified neither as ‘losers’ nor as ‘winners’, we tested whether they are specialized on intermediate land-use or abiotic levels, that is, whether they have an intermediate AWM with a narrower niche than expected. We standardized the niche breadth as weighted coefficient of variation (CV = AWSD/AWM) to account for the increase in SD with increasing mean, and compared observed CV and expected CV from the null models. This comparison allows us to distinguish ‘opportunists’ (observed CV ≥ expected CV) from species that are ‘specialized’ on intermediate land-use intensities (observed CV < expected CV and species not only occurring on one site, i.e., CV ≠ 0) [30]. The environmental niche (AWM, AWSD) and the assignment of low- and high-gradient specialists were also calculated for soil pH and soil moisture, although we did not adopt the ‘loser’/’winner’ terminology here unlike for land-use intensity.

### Species vulnerability

Vulnerability (classified as a rank variable comparable to IUCN categories: least concern, endangered to unknown extent, very rare, near threatened, critically endangered, endangered, vulnerable) of land snail species was obtained from the Red List 2011 (according to [56]; see Table 3). We tested the relation of vulnerability with the species’ habitat association by calculating the proportional occurrence in either forest or grassland habitats of a certain species’ presence; a ‘specialist’ was defined if more than 90% of all individuals found were present in one habitat (forest or grassland). The relation between vulnerability and species’ habitat association was tested by a linear model using the land-use management components and the abiotic conditions as fixed factors and the proportional occurrence as explanatory factor.

To further test if a species’ vulnerability can be predicted by its land-use response (‘winner’ or ‘loser’ status) and its relation to abiotic soil conditions, we used a general linearized model with Poisson distribution including vulnerability as response factor and the respective land-use parameter or abiotic factor, the number of plots where the species occurred and its total abundance as explanatory factors. Values for grazing and fertilization were square-root transformed prior to statistical analyses and data on abundances and occurrence were log transformed because of data structure.

Finally, we calculated a five-dimensional niche hypervolume (consistent with Hutchinson's *n*‐dimensional niche concept) as a proxy for the total ‘niche breadth’ of each snail species by multiplying the abundance-weighted standard deviations (AWSD) of all three single land-use components as well as of pH and soil moisture, respectively. The hypervolume was defined for forests and grasslands separately.

Whether the total niche breadth can predict vulnerability was tested using a Spearman rank correlation between the vulnerability and the five-dimensional niche hypervolume.

## Supplementary Information

**Additional file 1: Appendix 1.** Summary of significant effects of land-use parameters and abiotic factors in forests (forest management index Formi, proportion of non-native tress, proportion of dead wood with saw cuts, proportion of wood harvested, pH and soil moisture) and grasslands (land-use index LUI, mowing, grazing, fertilization, pH and soil moisture) on the community weighted mean of the maximum shell size, the number of offspring, light preference, humidity preference, drought resistance and inundation tolerance. * *p* < 0.05, ** *p* < 0.01, *** *p* < 0.001. ↓ negative effect, ↑ positive effect.

**Additional file 2: Appendix 2.** Influence of the abundance-weighted mean (AWM) of the forest management index on the maximum shell size, number of offspring, light preference, humidity preference, drought resistance and inundation tolerance in forests. Species in italics are land-use “winners”, species in bold are land-use “losers”.

**Additional file 3: Appendix 3.** Influence of the abundance-weighted mean (AWM) of the proportion of non-native trees on the maximum shell size, number of offspring, light preference, humidity preference, drought resistance and inundation tolerance in forests. Species in italics are land-use “winners”, species in bold are land-use “losers”.

**Additional file 4: Appendix 4.** Influence of the abundance-weighted mean (AWM) of the proportion of deadwood with saw cuts on the maximum shell size, number of offspring, light preference, humidity preference, drought resistance and inundation tolerance in forests. Species in italics are land-use “winners”, species in bold are land-use “losers”.

**Additional file 5: Appendix 5.** Influence of the abundance-weighted mean (AWM) of the proportion of wood harvested on the maximum shell size, number of offspring, light preference, humidity preference, drought resistance and inundation tolerance in forests. Species in italics are land-use “winners”, species in bold are land-use “losers”.

**Additional file 6: Appendix 6.** Influence of the abundance-weighted mean (AWM) of soil pH on the maximum shell size, number of offspring, light preference, humidity preference, drought resistance and inundation tolerance in forests. Species in italics are land-use “winners”, species in bold are land-use “losers”.

**Additional file 7: Appendix 7.** Influence of the abundance-weighted mean (AWM) of soil moisture on the maximum shell, size number of offspring, light preference, humidity preference, drought resistance and inundation tolerance in forests. Species in italics are land-use “winners”, species in bold are land-use “losers”.

**Additional file 8: Appendix 8.** Influence of the abundance-weighted mean (AWM) of land-use intensity (LUI) on the maximum shell size, number of offspring, light preference, humidity preference, drought resistance and inundation tolerance in grasslands. Species in italics are land-use “winners”, species in bold are land-use “losers”.

**Additional file 9: Appendix 9.** Influence of the abundance-weighted mean (AWM) of mowing on the maximum shell size, number of offspring, light preference, humidity preference, drought resistance and inundation tolerance in grasslands. Species in italics are land-use “winners”, species in bold are land-use “losers”.

**Additional file 10: Appendix 10.** Influence of the abundance-weighted mean (AWM) of grazing on the maximum shell size, number of offspring, light preference, humidity preference, drought resistance and inundation tolerance in grasslands. Species in italics are land-use “winners”, species in bold are land-use “losers”.

**Additional file 11: Appendix 11.** Influence of the abundance-weighted mean (AWM) of fertilization on the maximum shell size, number of offspring, light preference, humidity preference, drought resistance and inundation tolerance in grasslands. Species in italics are land-use “winners”, species in bold are land-use “losers”.

**Additional file 12: Appendix 12.** Influence of the abundance-weighted mean (AWM) of soil pH on the maximum shell size, number of offspring, light preference, humidity preference, drought resistance and inundation tolerance in grasslands. Species in italics are land-use “winners”, species in bold are land-use “losers”.

**Additional file 13: Appendix 13.** Influence of the abundance-weighted mean (AWM) of soil moisture on the maximum shell size, number of offspring, light preference, humidity preference, drought resistance and inundation tolerance in grasslands. Species in italics are land-use “winners”, species in bold are land-use “losers”.

**Additional file 14: Appendix 14.** Relation of the abundance-weighted means (AWM) of the forest management index, proportion of non-native trees, proportion of dead wood with saw cuts, proportion of wood harvested, pH and soil moisture and the proportional occurrence of a certain species in forests.

**Additional file 15: Appendix 15.** Relation of the abundance-weighted means (AWM) of the land-use intensity, mowing, grazing, fertilization, pH and soil moisture and the proportional occurrence of a certain species in forests.

## Data Availability

Snail data obtained by [27] and used in this study are available online under https://www.bexis.uni-jena.de/PublicData/PublicDataSet.aspx?DatasetId=24986. Data on snail vulnerability were obtained from the Red List 2011 according to [43] and snail traits were extracted from [38]. Environmental data and those for land-use intensity in grasslands and forests were obtained from the BExIS database (see Table 6).
