# Supplementary material for: Narrow environmental niches predict land-use responses and vulnerability of land snail assemblages
Source: BMC Ecol Evol. 2021 Feb 1;21:15. doi: 10.1186/s12862-020-01741-1 (PMC7853316; doi:10.1186/s12862-020-01741-1)
Supplement: Supplementary file 1 — Additional file 1: Appendix 1. Summary of significant effects of land-use parameters and abiotic factors in forests (forest management index Formi, proportion of non-native tress, proportion of dead wood with saw cuts, proportion of wood harvested, pH and soil moisture) and grasslands (land-use index LUI, mowing, grazing, fertilization, pH and soil moisture) on the community weighted mean of the maximum shell size, the number of offspring, light preference, humidity preference, drought resistance and inundation tolerance. * p < 0.05, ** p < 0.01, *** p < 0.001. ↓ negative effect, ↑ positive effect. [file 12862_2020_1741_MOESM1_ESM.pdf]

Appendix 1. Summary of significant effects of land-use parameters and abiotic factors in forests (forest management index Formi, proportion of non-native tress, proportion of dead wood with saw cuts, proportion of wood harvested, pH and soil moisture) and grasslands (land-use index LUI, mowing, grazing, fertilization, pH and soil moisture) on the community weighted mean of the maximum shell size, the number of offspring, light preference, humidity preference, drought resistance and inundation tolerance. * $p < 0.05$, ** $p < 0.01$, *** $p < 0.001$. ↓ negative effect, ↑ positive effect.

**Community weigthed mean of:**		**Swabian Alb**	**Hainich**	**Schorfheide**
Maximum shell size	Formi		↑***	↓***
	Proportion of non-native trees	↓***	↑***	↓*
	Proportion of dead wood with saw cuts	↓***	↑*	↓***
	Proportion of wood harvested	↓***	↑**	
	pH	↓**	↓***	↓***
	Soil moisture			↓***
Number of offspring	Formi		↑**	
	Proportion of non-native trees		↑***	↑*
	Proportion of dead wood with saw cuts	↓***		
	Proportion of wood harvested			
	pH		↓***	↑***
	Soil moisture		↓*	↑*
Light preference	Formi			↑***
	Proportion of non-native trees		↑***	↑***
	Proportion of dead wood with saw cuts	↓**		
	Proportion of wood harvested			
	pH		↑*	↑*
	Soil moisture	↑**		
Humidity preference	Formi	↓*	↓***	↑***
	Proportion of non-native trees	↓***	↓***	↑***
	Proportion of dead wood with saw cuts	↑***	↓**	↓***
	Proportion of wood harvested	↑**	↓*	
	pH		↓***	↑***
	Soil moisture	↑***		
Drought resistance	Formi		↑*	↓***
	Proportion of non-native trees	↑***	↑**	↓***
	Proportion of dead wood with saw cuts	↓***		
	Proportion of wood harvested	↓***		
	pH		↑*	↑*
	Soil moisture	↓***		
Inundation tolerance	Formi	↑***	↑*	
	Proportion of non-native trees	↑***	↑***	
	Proportion of dead wood with saw cuts	↑***		
	Proportion of wood harvested	↑***		↓**
	pH	↑***	↑***	↑*
	Soil moisture	↓**		↑**

**Community weigthed mean of:**		**Swabian Alb**	**Hainich**	**Schorfheide**
Maximum shell size	LUI			↓***
	Mowing	↓*		↓*
	Grazing			
	Fertilization	↑*		
	pH	↓**	↓**	↓***
	Soil moisture	↓*	↓**	
Number of offspring	LUI	↑*	↑***	↓***
	Mowing			↑***
	Grazing	↓***	↑*	↓***
	Fertilization			↓*
	pH			↑***
	Soil moisture			↑***
Light preference	LUI	↑*	↑**	
	Mowing			↑***
	Grazing	↓***	↑***	↓***
	Fertilization	↓**		
	pH		↑***	
	Soil moisture		↑*	↑*
Humidity preference	LUI	↑*	↑*	↓***
	Mowing			
	Grazing	↓*		↓***
	Fertilization			↓**
	pH		↓**	↑***
	Soil moisture	↓*		↑***
Drought resistance	LUI			↑***
	Mowing	↓***	↓**	↑**
	Grazing	↓***		↓**
	Fertilization		↓**	
	pH		↑*	
	Soil moisture	↓**	↑***	
Inundation tolerance	LUI			↓***
	Mowing			
	Grazing	↓***	↑***	↓**
	Fertilization	↓**		↓**
	pH	↑**	↑***	↑***
	Soil moisture	↑***	↑***	↑***
