# Supplementary material for: Narrow environmental niches predict land-use responses and vulnerability of land snail assemblages
Source: BMC Ecol Evol. 2021 Feb 1;21:15. doi: 10.1186/s12862-020-01741-1 (PMC7853316; doi:10.1186/s12862-020-01741-1)

Appendix 04

Influence of the abundance-weighted mean (AWM) of the proportion of deadwood with saw cuts on the maximum shell size number of offspring, light preference, humidity preference, drought resistance and inundation tolerance in forests. Species in italics are land-use “winners”, species in bold are land-use “losers”.

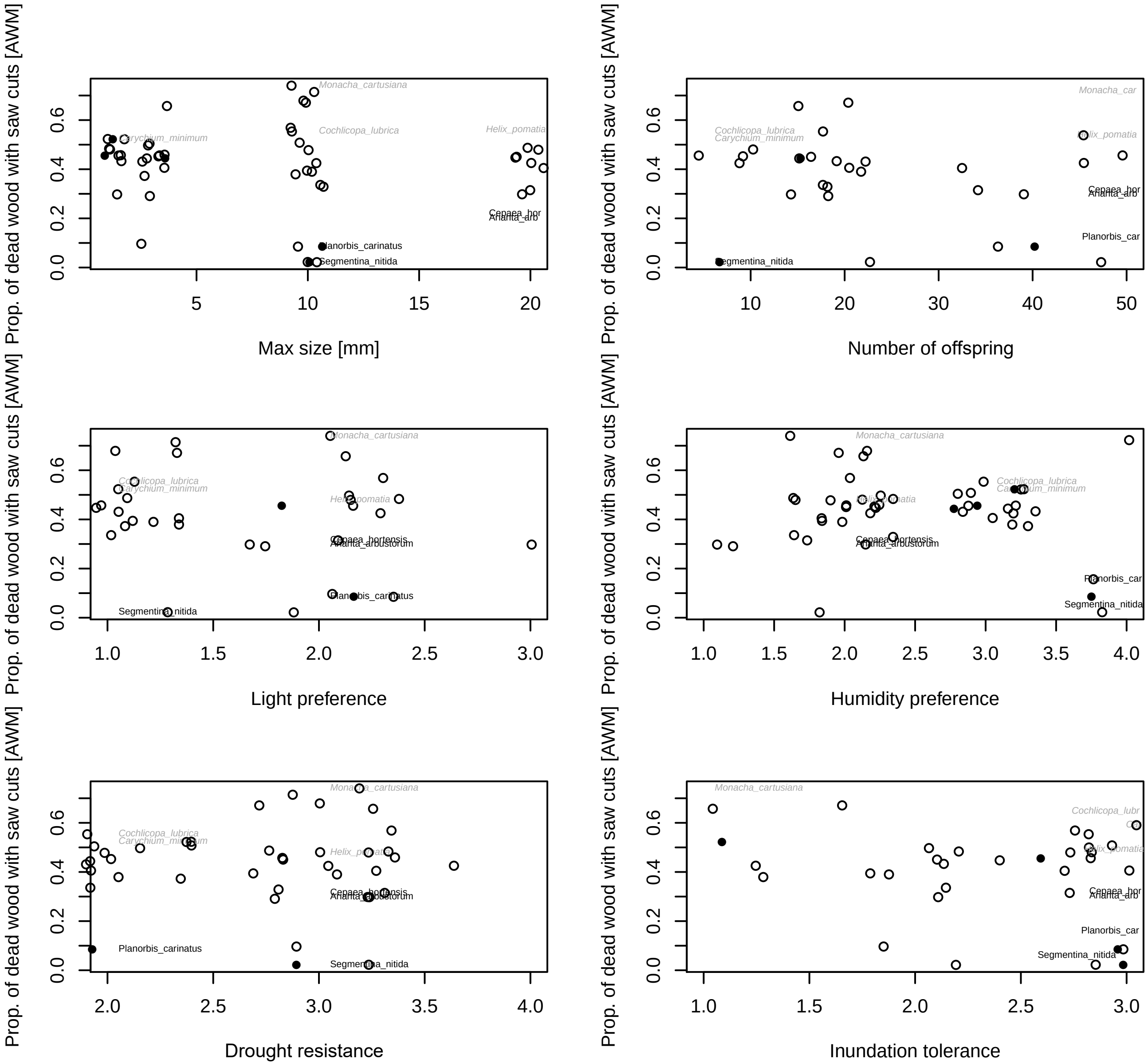

Supplement: Supplementary file 4 — Additional file 4: Appendix 4. Influence of the abundance-weighted mean (AWM) of the proportion of deadwood with saw cuts on the maximum shell size, number of offspring, light preference, humidity preference, drought resistance and inundation tolerance in forests. Species in italics are land-use “winners”, species in bold are land-use “losers”. [file 12862_2020_1741_MOESM4_ESM.pdf]
