# Supplementary material for: Narrow environmental niches predict land-use responses and vulnerability of land snail assemblages
Source: BMC Ecol Evol. 2021 Feb 1;21:15. doi: 10.1186/s12862-020-01741-1 (PMC7853316; doi:10.1186/s12862-020-01741-1)

Appendix 09

Influence of the abundance-weighted mean (AWM) of mowing on the maximum shell size number of offspring, light preference, humidity preference, drought resistance and inundation tolerance in grasslands. Species in italics are land-use “winners”, species in bold are land-use “losers”.

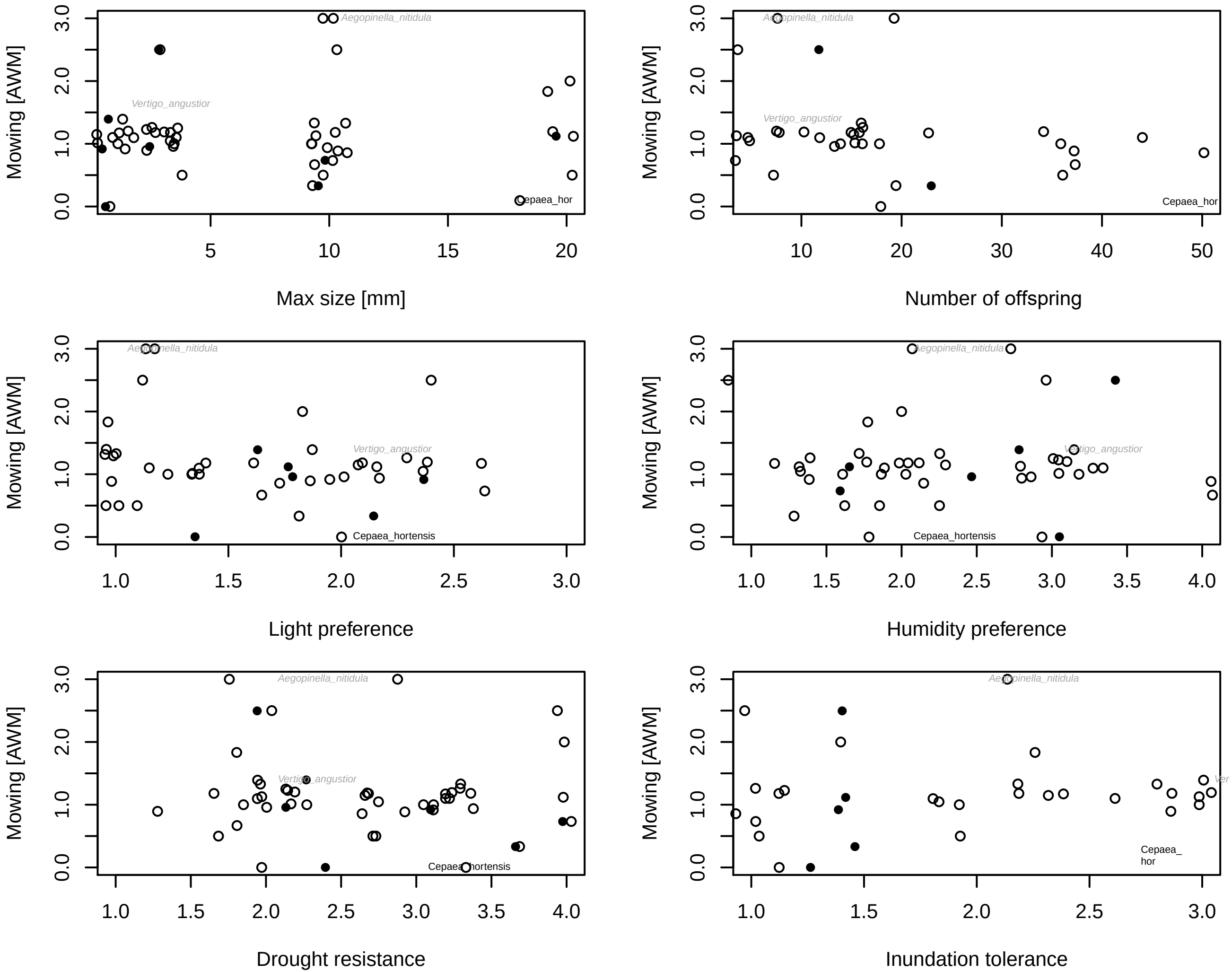

Supplement: Supplementary file 9 — Additional file 9: Appendix 9. Influence of the abundance-weighted mean (AWM) of mowing on the maximum shell size, number of offspring, light preference, humidity preference, drought resistance and inundation tolerance in grasslands. Species in italics are land-use “winners”, species in bold are land-use “losers”. [file 12862_2020_1741_MOESM9_ESM.pdf]
