# Supplementary material for: Narrow environmental niches predict land-use responses and vulnerability of land snail assemblages
Source: BMC Ecol Evol. 2021 Feb 1;21:15. doi: 10.1186/s12862-020-01741-1 (PMC7853316; doi:10.1186/s12862-020-01741-1)

Appendix 14

Relation of the abundance-weighted means (AWM) of the forest management index, proportion of non-native trees, proportion of dead wood with saw cuts, proportion of wood harvested, pH and soil moisture and the proportional occurrence of a certain species in forests.

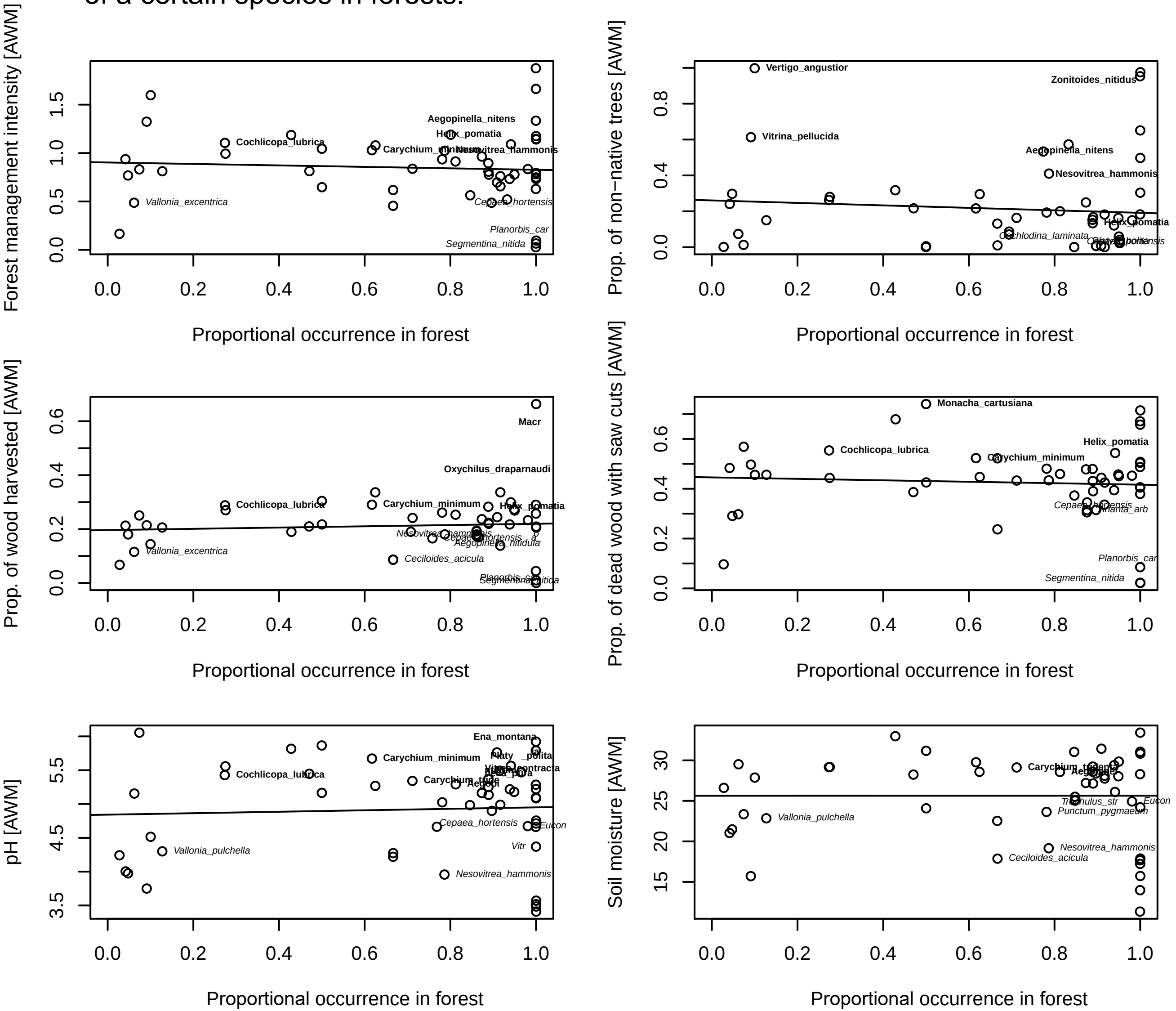

Supplement: Supplementary file 14 — Additional file 14: Appendix 14. Relation of the abundance-weighted means (AWM) of the forest management index, proportion of non-native trees, proportion of dead wood with saw cuts, proportion of wood harvested, pH and soil moisture and the proportional occurrence of a certain species in forests. [file 12862_2020_1741_MOESM14_ESM.pdf]
