# Supplementary material for: Narrow environmental niches predict land-use responses and vulnerability of land snail assemblages
Source: BMC Ecol Evol. 2021 Feb 1;21:15. doi: 10.1186/s12862-020-01741-1 (PMC7853316; doi:10.1186/s12862-020-01741-1)

Appendix 15

Relation of the abundance-weighted means (AWM) of the land-use intensity, mowing, grazing, fertilization, pH and soil moisture and the proportional occurrence of a certain species in forests.

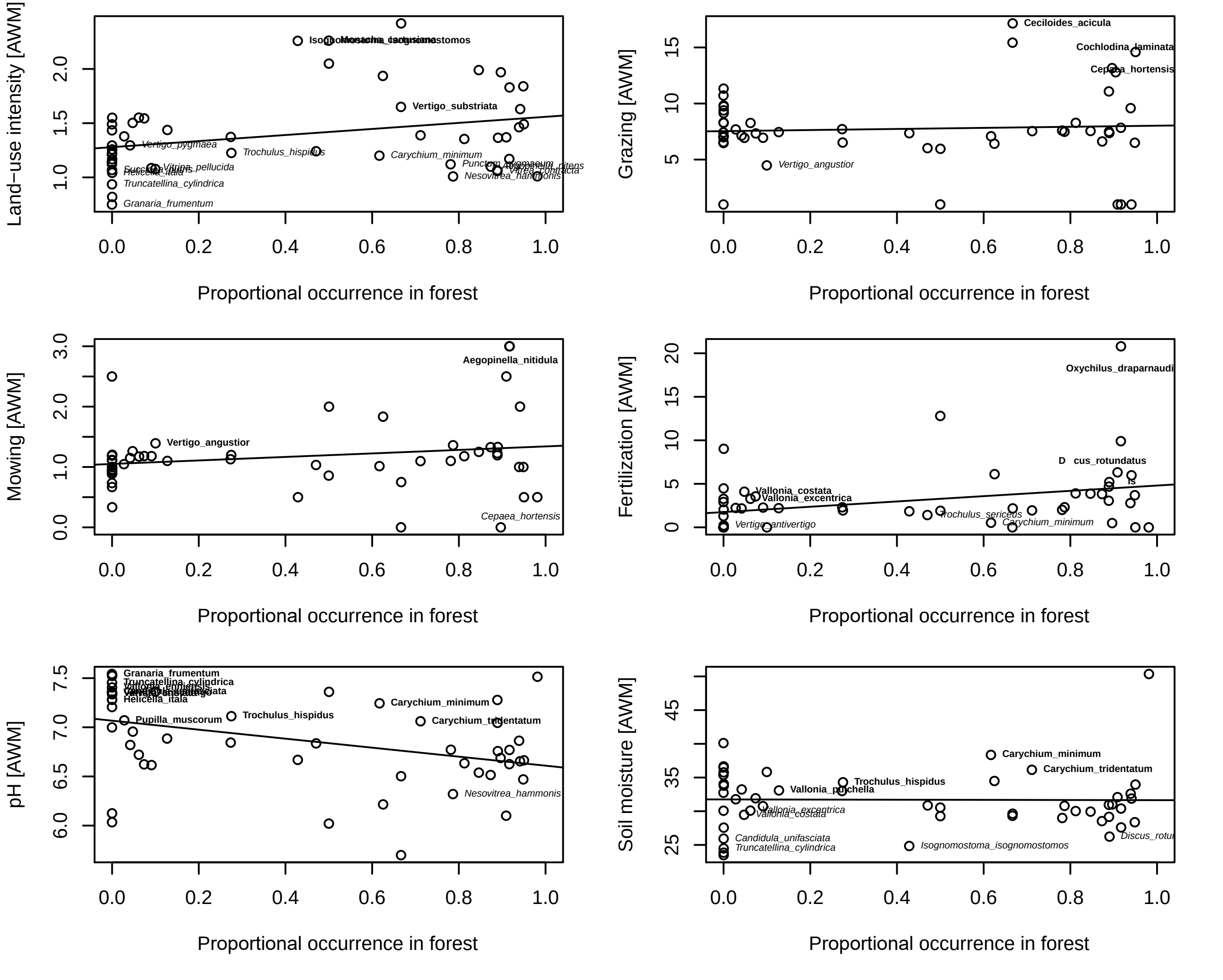

Supplement: Supplementary file 15 — Additional file 15: Appendix 15. Relation of the abundance-weighted means (AWM) of the land-use intensity, mowing, grazing, fertilization, pH and soil moisture and the proportional occurrence of a certain species in forests. [file 12862_2020_1741_MOESM15_ESM.pdf]
